# Identification of Co-Circulating Dengue and South America–Origin Zika Viruses, Pakistan, 2021–2022

**DOI:** 10.3201/eid3108.250342

**Published:** 2025-08

**Authors:** Najeeha T. Iqbal, Kaitlin Sawatzki, Kumail Ahmed, Jennifer Tisoncik-Go, Elise Smith, Kathleen Voss, John Cornelius, Lu Wang, Alicen B. Spaulding, Leonid Serebryannyy, Daniel C. Douek, Muhammad Asif Syed, Syed Faisal Mahmood, Erum Khan, Wesley C. Van Voorhis, Michael Gale

**Affiliations:** The Aga Khan University, Karachi, Pakistan (N.T. Iqbal, K. Ahmed, S.F. Mahmood, E. Khan); University of Washington, Seattle, Washington, USA (K. Sawatzki, J. Tisoncik-Go, E. Smith, K. Voss, J. Cornelius, W.C. Van Voorhis); National Institutes of Health, National Institute of Allergy and Infectious Diseases, Bethesda, Maryland, USA (L. Wang, A.B. Spaulding, L. Serebryannyy, D.C. Douek); Pakistan Field Epidemiology Laboratory Training Program, Karachi (M.A. Syed); University of Minnesota, Minneapolis, Minnesota, USA (M. Gale, Jr.)

**Keywords:** Dengue virus, Zika virus, flavivirus, biosurveillance, molecular epidemiology, genetics, metagenomics, viral RNA

## Abstract

We collected samples from febrile patients in Karachi, Pakistan, in 2021–2022. Sequencing, molecular, and serologic screens revealed dengue serotype 2 and Zika virus. The Zika lineage was inferred to be from Brazil in 2016, indicating unobserved circulation. We conclude that Zika virus contributes to perceived dengue outbreak burden in Pakistan.

*Orthoflavivirus* is a genus of arthropod-borne, positive-strand RNA viruses capable of causing serious disease outbreaks in humans (*1*). Dengue virus (DENV) and Zika virus (ZIKV) are clinically relevant species with widespread circulation in tropical and subtropical climates via *Aedes* spp. mosquitoes; ≈400 million persons are at risk for infection each year (*2*–*5*). In 2018, Rajasthan State in India reported the country’s first known cases of ZIKV infection (*6*). In that report, researchers tested household contacts and unrelated febrile persons near a single index case using a quantitative real-time reverse transcription PCR (qRT-PCR). The authors found that 153 (7.48%) of 2,043 contacts were positive for ZIKV viral RNA. This result demonstrates that ZIKV can begin circulation in new regions without a specific outbreak event. We report evidence of co-circulation of dengue virus serotype 2 (DENV-2) and Brazil-origin ZIKV in Pakistan.

## The Study

In November 2021, local news sources reported an unknown viral outbreak in Karachi, Pakistan, which was subsequently investigated by the Field Epidemiology Lab Training Program of the Health Department–Sindh (*7*–*9*). DENV is endemic to the region, and infection outbreaks are common. We selected 7 patients with symptoms consistent with arbovirus infection, including fever, chills, headache and myalgia, for metagenomic analysis. We also probed blood samples with a pan-virus oligo panel and sequenced the samples to identify possible causative pathogens (Appendix, Tables 4, 5). We identified several viruses, including pegivirus, DENV), and ZIKV (Appendix Table 6). Results confirmed 6 patients were positive for DENV-2 and 2 were positive for ZIKV, including 1 person (patient E) co-infected with both viruses. Those results were consistent with qRT-PCR, apart from a weakly positive DENV that we could not identify in patient F from metagenomic reads (Table 1).

**Table 1:** Real time qRT-PCR results for DENV and ZIKV in metagenomics analysis groups for co-circulation of dengue and South America–origin Zika viruses, Pakistan, 2021–2022

Patient	DENV (Ct<40)	ZIKV (Ct<40)	ZIKV (Ct<38.5)	ZIKV (Ct<38.5)
A	**20.3**	Negative	Not done	Not done
B	**39.74**	Negative	Not done	Not done
C	**26.91**	Negative	Not done	Not done
D	**31.74**	Negative	Not done	Not done
E	**37.77**	**38.73**	Not done	Not done
F	**39.35**	Negative	**37.33**	39.05
G	Not done	Not done	Not done	Not done

*Bold text represents positive assay values. Ct, cycle threshold; DENV, dengue virus; qRT-PCR, quantitative reverse transcription PCR; ZIKV, Zika virus.

We obtained 7 complete and 1 partial orthoflavivirus genomes from the 7 patient samples (Appendix). Phylogenetic analysis confirmed all observed DENV to be the cosmopolitan genotype of DENV-2 of recent East and Southeast Asia origin (Appendix Figure 7). However, the 2 ZIKV strains we observed were more closely related to ZIKV circulating in South America than to contemporaneous ZIKV in neighboring countries to Pakistan (Figure, panels A, B; Appendix Figure 8). Three ZIKV amino acid changes were unique to the Pakistan viruses, and their inferred closest ancestor was most closely comparable to the Brazil ZIKV subclade (Figure, panel C). We observed 2 changes in prM, T74A and S109P. S109P is located in the prM region, which maps to the binding interface with Env, and both changes straddle the prM host cleavage site. We noted the third amino acid change, K587R, located at the 3′ end of the NS3-coding region, adjacent to NS4A. We identified a final change at the 3′ end of the NS1-coding region encoding M349V that distinguishes the subclade among other viruses circulating in Brazil and other regions of South America (Appendix Figure 9).

**Figure:**
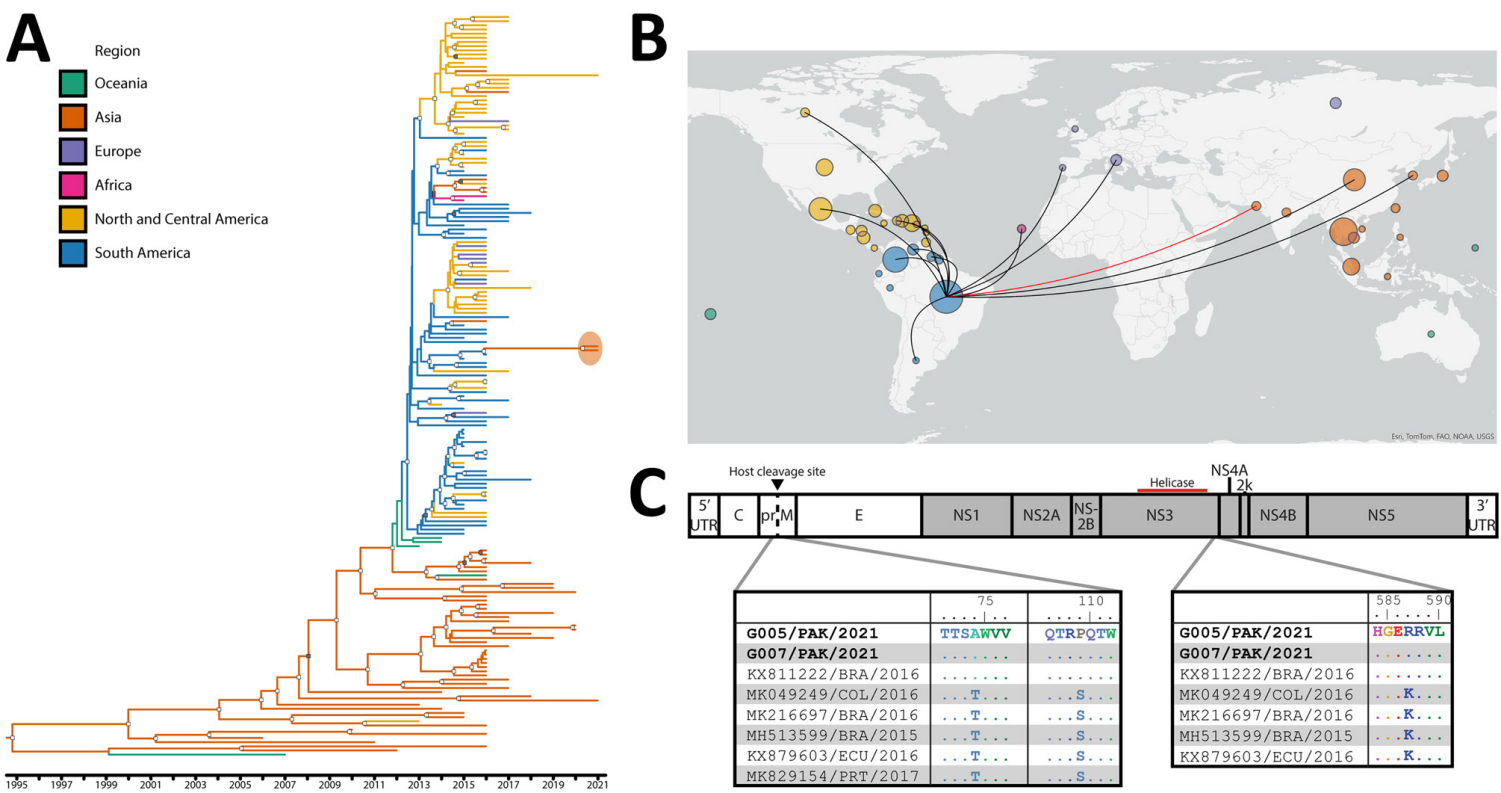
Phylogenetic analysis of Zika viruses (ZIKV) identified in a study of co-circulation of dengue and South American–origin Zika viruses, Pakistan, 2021–2022. A) BEAST (https://beast.community/index.html) time-aware maximum clade credibility tree describing inferred genetic lineage of global Asian-lineage ZIKV, colored by observed and estimated geographic origin. Branch backbones are colored when called with >80% confidence by Augur. Two newly described viruses from Pakistan are circled in orange, with the closest observed ancestors derived from circulating South American ZIKV. Open circles indicate posterior probability >0.9, solid dots 0.8–0.9. B) Phylogeographic map illustrating inferred international ZIKV transmission events originating in Brazil from viruses included in panel A. Map was visualized by inferred origin and transmission using Auspice (https://docs.nextstrain.org/projects/auspice/en/stable/index.html). Circle size is relative to the number of included viruses from the country and colored by continent. Red line highlights the inferred Brazil to Pakistan incursion. C) ZIKV sequences selected from the same clade and subclade as Pakistan-origin viruses aligned to G005/PAK/2021. Identical amino acid residues are shown as dots. We identified 2 changes in prM, T74A and S109P (left), and 1 in NS3, K587R (right). G005/PAK/2021 corresponds to patient E; G007/PAK/2021 corresponds to patient F. NS, nonstructural; UTR, untranslated region.

In 2022, we collected samples from a different cohort of 13 patients with evidence of ZIKV antibodies. Those patients demonstrated symptoms characteristic of arbovirus infection, including fever, rash, arthralgia, and thrombocytopenia (Appendix Table 7). We collected blood samples on day 1 (n = 4) and day 28 (n = 12) after hospital admission and assayed for evidence of recent DENV and ZIKV infection by ELISA and Meso Scale discovery (MSD) immunoassays (Table 2; Appendix). We tested patients with >1 positive or equivocal Zika IgG ELISA result by qRT-PCR for evidence of active infection. All 4 patient samples from day 1 tested positive for DENV by qRT-PCR; 1 patient (patient 3) tested positive for ZIKV infection by duplex qRT-PCR. By day 28, two of the 12 patient samples we collected were positive for DENV and none were positive for ZIKV by qRT-PCR. Three patients were antibody positive for ZIKV by both ELISA and MSD, which strictly controls for cross-reactivity between DENV-2 and ZIKV nonstructural (NS) 1 proteins.

**Table 2:** Real time qRT-PCR and serologic assay results for DENV and ZIKV infection determination in study of co-circulation of dengue and South America–origin Zika viruses, Pakistan, 2021–2022*

Patient	Day	DENV (Ct<40)	ZIKV (Ct<40)	ZIKV (Ct<38.5)	ZIKV (Ct<38.5)	Zika IgG ELISA (OD)	MSD Assay IgG, ECL (ZIKV/DENV)
1	1	**24.88**	Not done	Negative	**37.57**	**0.2269**	5,572/9,031 (0.6×)
2	1	**32.95**	Not done	Negative	Negative	0.1356 (eq)	46,364/69,634 (0.6×)
3	1	**38.59**	Negative	**34.72**	**37.46**	0.1855 (eq)	35,809/57,433 (0.6×)
4	1	**37.64**	Negative	Negative	**37.21**	**1.9765**	**42,482/5,103 (8×)**
5	28	Negative	Not done	Negative	Negative	**2.1297**	103,454/378,372 (0.2×)
6	28	Negative	Not done	Negative	Negative	**1.1431**	25,304/111,319 (0.2×)
2	28	Negative	Not done	**33.36**	Negative	**2.3136**	167,354/644,477 (0.2×)
7	28	Negative	Not done	Negative	Negative	**1.192**	24,500/115,362 (0.2×)
8	28	**39.23**	Not done	Negative	Negative	**0.2008**	**14,599/5,609 (2.6×)**
9	28	**39.50**	Not done	Negative	Negative	**0.8356**	36,655/1,098,636 (0.03×)
3	28	Negative	Negative	**38.44**	38.93	**1.5498**	**30,842/5,012 (6×)**
4	28	Negative	Negative	Not done	Not done	−0.0012	8,975/319,294 (0.03×)
10	28	Negative	Not done	Negative	Negative	**0.2963**	22,245/413,868 (0.05×)
11	28	Negative	Not done	Negative	Negative	0.1616 (eq)	9,915/462,840 (0.02×)
12	28	Negative	Not done	Negative	39.04	**0.2974**	75,115/194,188 (0.4×)
13	28	44.26	Not done	**36.47**	Negative	**1.3555**	53,643/161,184 (0.3×)

*Bold text represents positive assay values. Ct, cycle threshold; DENV, dengue virus; ECL, electrochemiluminescence; eq, equivocal; MSD, Meso Scale Discovery; OD, optical density; qRT-PCR, quantitative reverse transcription PCR; ZIKV, Zika virus.

Among the 2022 cohort of patients we sampled, 2 patients had longitudinal samples consistent with DENV and ZIKV co-infection. The first (patient 3) was symptomatic for 7 days upon admission. He initially sought treatment for respiratory symptoms, and tests revealed an elevated total leukocyte count (22.8 × 10^9^ cells/L; reference range 5.0–10.0 × 10^9^ cells/L), with neutrophils comprising 88% (reference range 50–80%) and lymphocytes 7% (reference range 20–40%). On day 1 of inpatient treatment, he tested positive by qRT-PCR for DENV (cycle threshold [Ct] 38.59) and ZIKV (Cts 34.72, 37.46) co-infection and was equivocal for ZIKV antibodies (Table 2). By day 28, he had ZIKV NS1 IgG as measured by ELISA (optical density [OD] 1.5498) and MSD, which showed a 6-fold higher ZIKV signal in the ZIKV/DENV-2 NS1 IgG ratio (electrochemiluminescence [ECL] 30,842/5,012).

The second co-infected patient (patient 4) had been symptomatic for 3 days before hospital admission. His symptoms were consistent with dengue fever, including a low platelet count (38 × 10^9^/L; reference range 150–400 × 10^9^/L) and a high lymphocyte percentage (47.9%; reference range 20–40%). On day 1 of inpatient treatment, he tested positive by both ELISA (OD 1.9765) and MSD (ECL 42,482/5,103, 8-fold) for ZIKV antibodies. qRT-PCR of this patient’s sample was initially positive for DENV and equivocal for ZIKV (1/2 positive amplicons). By day 28, there was a major peak in the DENV-2 NS1 IgG response measured by MSD assay, with a corresponding decline in ZIKV IgG (ECL 8,975/319,294, 0.03-fold). Those results are suggestive of an initial, symptomatically mild ZIKV infection followed by emergent DENV co-infection. Both co-infected patients, as well as patient E, from whom we assembled both viral genomes, represent 3 cases of probable ZIKV-DENV co-infection.

## Conclusions

Identifying and characterizing etiologic agents associated with infections of unknown etiology in Pakistan is critical to understanding the consequences of new or re-emerging viruses in the region. We identified unexpected ZIKV in Pakistan using a panviral metagenomics approach and were able to confirm it in additional samples using a real time qRT-PCR. Antibody testing further revealed co-circulation contemporaneous with dengue virus, with high seroconversion. 

Metagenomic sequencing further revealed ZIKV as an arbovirus importation into the region. Rather than originating from bordering or nearby countries, the most closely related available ZIKV sequences originate from Brazil in 2016. This distinct clade of Asian-genotype ZIKV emerged in Brazil in 2015 and rapidly spread across the Americas. Brazil-origin ZIKV from the same time period was exported to many other countries, including Italy, South Korea, and Cabo Verde (Figure, panel C). Although many of those events are self-limiting, favorable ecologic conditions can establish new areas of virus circulation, now making ZIKV a pathogen that should be part of both public health guidelines and private practice diagnostic considerations in Pakistan.

In summary, the evidence revealed from our investigation indicates that Brazil-origin ZIKV has spread to local *Aedes* spp. mosquitoes and is endemically circulating in Pakistan. ZIKV and DENV overlap in host mosquito species (*Ae. aegypti* and *Ae. albopictus*); therefore, incorporating ZIKV screening and surveillance in DENV management programs would make sense. As new arboviruses are discovered, outbreaks across diverse, international geographic areas will prompt the need to interrogate acute and convalescent samples to identify causative agents and develop specific diagnostic and therapeutic strategies for use in outbreak responses.

Appendix Additional information for identification of co-circulating dengue and South American–origin Zika viruses, Pakistan, 2021–2022
