# Supplementary material for: Identification of Co-Circulating Dengue and South America–Origin Zika Viruses, Pakistan, 2021–2022
Source: Emerg Infect Dis. 2025 Aug;31(8):1648–51. doi: 10.3201/eid3108.250342 (PMC12309745; doi:10.3201/eid3108.250342)
Supplement: Appendix — Additional information for identification of co-circulating dengue and South American–origin Zika viruses, Pakistan, 2021–2022 [file 25-0342-Techapp-s1.pdf]

Article DOI: http://doi.org/10.3201/eid3108.250342

EID cannot ensure accessibility for supplementary materials supplied by authors. Readers who have difficulty accessing supplementary content should contact the authors for assistance.

Identification of Co-Circulating Dengue and South America–Origin Zika Viruses, Pakistan, 2021–2022

Appendix

Methods

Study population

Samples were collected from 20 patients in two cohorts with suspected arbovirus disease admitted to Aga Khan Hospital or a private hospital in Karachi, Pakistan between November 2021 and October 2022 (Appendix Figure 1). Enrollment, sample collection and processing protocols were approved by the Ethical Review Committee of Aga Khan University (ERC#4794). For the metagenomic cohort, inclusion criteria were patients presenting with febrile illness, 1 week of fever history, and a negative Rapid Dengue NS1 test. Patients younger than 1 or older than 75 years were excluded. Seven patients were sequenced and included in this study. For the serology cohort, patients were selected from an ongoing study administered by United World Antiviral Research Network (UWARN) that investigates fevers of unknown origin. Thirteen patients with evidence of Zika virus infection by Zika IgG ELISA were included for additional testing in this study.

Sample collection and processing

The Field Epidemiology Lab Training Program (FELTP), administered by the provincial Health Department in Sindh, and Aga Khan University Hospital-Pakistan center for UWARN coordinated sample collection and processing. Blood samples were collected in EDTA tubes and inverted to mix. Tubes were centrifuged 1000 g for 10 minutes at 4°C and the plasma layer aliquoted. Samples were shipped on dry ice to Pandemic Response Repository through Microbial and Immune Surveillance and Epidemiology (PREMISE) at NIAID for serology. RNA was

extracted using the QIAamp Viral RNA Extraction Kit (Qiagen, https://www.qiagen.com) according to the manufacture's protocol and shipped on dry ice to the University of Washington for sequencing.

Pan-viral metagenomics sequencing

Samples were stored at −80°C until processing in BSL2+ with BSL3 practices. RNA integrity was evaluated using an Agilent 2100 Bioanalyzer or 4200 TapeStation (Agilent Technologies, https://www.agilent.com). cDNA was generated with Random Primer 6 and the ProtoScript II First Strand cDNA synthesis kit [New England Biolabs (NEB), https://www.neb.com]. The second strand was synthesized using the NEBNext® Ultra II Non-Directional RNA Second Strand Synthesis Module (NEB). Library preparation was completed using the Twist Total Nucleic Acids Library Preparation EF Kit 2.0 and Twist UDI Primers (Twist Biosciences, https://www.twistbioscience.com). Bead-purified libraries were checked using the Bioanalyzer High Sensitivity DNA assay (Agilent) and pooled up to 8-plex, with the total pool never exceeding 1500 ng. Indexed pools were hybridized at 70°C for 16 hours with the Twist Comprehensive Viral Research Panel (Twist Bioscience) and amplified using KAPA HiFi HotStart ReadyMix (Roche, https://www.roche.com). Enriched libraries were bead purified, validated with the Bioanalyzer High Sensitivity DNA kit (Agilent) and multiplexed. Samples were sequenced to ≈10 million total reads using the Illumina NextSeq 500/550 High Output v2 Sequencing Kit (150 cycles) (Illumina, https://illumina.com).

Viral metagenomics

Raw sequence data were assessed for quality using Illumina's BaseSpace platform and FastQC (v0.11.8). Sequence adapters and low-quality reads were trimmed with Trimgalore (v0.6.4). Specificity of the Twist pan-viral approach was tested using iterative subsampling and mapping to DENV2 reference genome for positive and negative control patient samples (Appendix Figure 6). The assay and sequencing depth were appropriate for high confidence calls, including differentiation of false positives with low genome coverage. Sequences were processed with Genome Detective (https://www.genomedetective.com)(v2.48) (*1*). For samples with high confidence DENV or ZIKV calls, trimmed reads were mapped to reference DENV2 (NC_001474.2) and ZIKV (NC_035889.1) using BWA (v0.7.17) and consensus called with Samtools (v1.15.1) and BCFtools (v1.10.2).

Phylogenetic analysis

Phylogenetic analysis was performed for assembled Zika (N = 2) and dengue 2 viruses (N = 6). All human- and mosquito-origin ZIKV and DENV-2 genomes with collection year and continent metadata were collected from BV-BRC (*2*). Sequences noted as lab or stock cultures, passaged multiple times, duplicates, including >10% Ns or with <90% genome coverage were removed resulting in 3936 DENV2 and 898 ZIKV genomes. Each UTR and gene was aligned using ClustalΩ (v1.2.4), trimmed, and concatenated. IQ-TREE (v1.6.12) (*3–5*) was used for preliminary maximum likelihood (ML) tree generation. ML trees were iteratively subset to preserve diversity with Treemmer (v0.3) (relative tree length of 90%). West African clade ZIKV was removed as new viruses were inferred to be the Asian clade. The final sequence was trimmed to n = 150 DENV2 and n = 176 ZIKV genomes (*6*) (Appendix Figures 2 and 3**)**.

Appropriateness for molecular clock models was tested on final subset ML trees with TempEst v1.5 using best-fitting root (Appendix Figure 4). For ZIKV, a null set of 1000 randomly scrambled metadata-genomes was processed (GTR+F+R3, ultrafast bootstrap = 1001), and the null distribution compared to the observed values (Appendix Figure 5). Clock and tree model selection was performed with BEAST (v1.10.4) using path sampling/stepping stone sampling marginal likelihood estimation with 100 path steps and 1 million chain length (Appendix Tables 1 and 2). Samples were time-stamped to their collection year with 1 year uncertainty. Final analyses were independently run 5–6 times on BEAST with a chain length of 200 million generations using a skyline tree prior, relaxed clock with an uncorrelated lognormal distribution, and GTR + 4Γ substitution model with empirical base frequencies. Tracer (v1.7) (*7*) was used to confirm convergence and ESS values over 200. Trees were combined with LogCombiner v1.10.4, 10% burn-in removed, and maximum clade credibility trees selected with TreeAnnotator v1.10.4. Final trees were visualized in FigTree v1.4. Geographic metadata were integrated with Augur and visualized using Auspice v2.56.0 and ArcGIS Pro v3.2.0.

Serologic assays

Indirect Enzyme-Linked Immunosorbent Assay (ELISA) was performed using the Human Anti-ZIKV IgG ELISA kit (R&D Systems, https://www.rndsystems.com) according to the manufacturer's recommendations. Plates were read immediately on a SpectraMax Paradigm (Molecular Devices, https://www.moleculardevices.com) at 450 nm with a correction wavelength of 540 nm. Results were normalized by subtracting the background plate OD reading

from the assay microplate OD values. A normalized OD >0.200 was considered a ZIKV-positive sample, 0.100 ≤OD ≥0.200 as equivocal, and OD <0.100 as ZIKV-negative.

Electrochemiluminescence assays for IgG against Zika and Dengue 2 NS1 protein were performed using the Meso Scale Discovery platform (MSD, https://www.mesoscale.com). Briefly, 384-well plates were coated overnight at 4°C with 1 µg/mL Zika NS1 or Dengue 2 NS1 (R&D Systems) in 1X phosphate-buffered saline. Plates were washed and blocked for 1 hour with shaking at room temperature. Following blocking, plates were washed and incubated with 1:100 diluted samples and controls for 1 hour with shaking at room temperature. Plates were washed and incubated with 1 µg/mL Sulfo-tag anti-human IgG (MSD) for 1 hour at room temperature with shaking. Plates were washed, 1X MSD read buffer applied, and read on a Meso Sector S600 (MSD). Sample signal from each Zika NS1 readout was divided by Dengue-2 NS1 readout to calculate relative binding enrichment.

Quantitative real time reverse transcription PCR (qRT-PCR)

Extracted RNA was tested using up to three qRT-PCR assays (Appendix Table 3). 1. Pan-DENV with multiplexed probes for the identification of dengue serotypes 1–4 (Biosearch Technologies, https://www.biosearchtech.com), 2. Pan-ZIKV with a single probe (Biosearch Technologies), and 3. Duplex ZIKV with two probes for distinct amplicons (IDT, https://www.idtdna.com). All primers and probes were diluted to 20 µM and 5 µM, respectively. Real time assays were performed using the Platinum Superscript III Invitrogen One-Step qRT-PCR kit (Thermo Fisher) and 5µL RNA template. qRT-PCR was performed using a CFX96 thermal cycler (Bio-Rad, https://www.bio-rad.com) at 52°C for 15 minutes; 94°C for 2 minutes; 45 cycles at 94°C for 15 seconds, 55°C for 20 seconds (*Acquisition), 60°C for 20 seconds; and a final extension at 68°C for 20 seconds. Each run included a non-template control and positive controls (BEI resources, https://www.beiresources.org).

Data availability

Genetic data has been deposited in open access databases. Viral genome accessions are (*1*): ZIKV full genomes, GenBank PQ066186–7 (*2*); DENV-2 full genomes, GenBank PQ069805–9; and (*3*) DENV-2, partial genome, GISAID EPI_ISL_19304156. Raw data are available at NCBI SRA SRR32520601–7.

References

1. Vilsker M, Moosa Y, Nooij S, Fonseca V, Ghysens Y, Dumon K, et al. Genome Detective: an automated system for virus identification from high-throughput sequencing data. Bioinformatics. 2019;35:871–3. PubMed https://doi.org/10.1093/bioinformatics/bty695

2. Olson RD, Assaf R, Brettin T, Conrad N, Cucinell C, Davis JJ, et al. Introducing the Bacterial and Viral Bioinformatics Resource Center (BV-BRC): a resource combining PATRIC, IRD and ViPR. Nucleic Acids Res. 2023;51(D1):D678–89. PubMed https://doi.org/10.1093/nar/gkac1003

3. Minh BQ, Schmidt HA, Chernomor O, Schrempf D, Woodhams MD, von Haeseler A, et al. IQ-TREE 2: New models and efficient methods for phylogenetic inference in the genomic era. Mol Biol Evol. 2020;37:1530–4. PubMed https://doi.org/10.1093/molbev/msaa015

4. Hoang DT, Chernomor O, von Haeseler A, Minh BQ, Vinh LS. UFBoot2: Improving the Ultrafast Bootstrap Approximation. Mol Biol Evol. 2018;35:518–22. PubMed https://doi.org/10.1093/molbev/msx281

5. Kalyaanamoorthy S, Minh BQ, Wong TKF, von Haeseler A, Jermiin LS. ModelFinder: fast model selection for accurate phylogenetic estimates. Nat Methods. 2017;14:587–9. PubMed https://doi.org/10.1038/nmeth.4285

6. Menardo F, Loiseau C, Brites D, Coscolla M, Gygli SM, Rutaihwa LK, et al. Treemmer: a tool to reduce large phylogenetic datasets with minimal loss of diversity. BMC Bioinformatics. 2018;19:164. PubMed https://doi.org/10.1186/s12859-018-2164-8

7. Suchard MA, Lemey P, Baele G, Ayres DL, Drummond AJ, Rambaut A. Bayesian phylogenetic and phylodynamic data integration using BEAST 1.10. Virus Evol. 2018;4:vey016. PubMed https://doi.org/10.1093/ve/vey016

Appendix Table 1. DENV model selection in BEAST using PS/SS MLE to investigate co-circulation of dengue and South American–origin Zika viruses, Pakistan, 2021–2022*

Clock	Tree	PS log MLE	SS log MLE	Rank
Relaxed	Skyline	−112129.573943	−112135.800803	1
Relaxed	Exponential	−112134.208748	−112137.035396	2
Relaxed	Skyride	−112158.659198	−112166.268221	3
Relaxed	Constant	−112169.031368	−112170.515417	4
Random	Skyride	−112241.642808	−112248.124269	5
Random	Skyline	−112250.854750	−112258.964391	6
Random	Exponential	−112444.491463	−112434.448338	7
Random	Constant	−112622.701123	−112623.790487	8
Strict	Skyline	−113240.105197	−113242.721905	9
Strict	Exponential	−113259.065631	−113257.723317	10
Strict	Skyride	−113288.997921	−113292.263829	11
Strict	Constant	−113302.365635	−113304.192591	12

*Path sampling (PS)/stepping stone (SS) marginal likelihood estimation (MLE); DENV: Dengue virus; Bayesian Evolutionary Analysis Sampling Trees (https://beast.community/index.html)

Appendix Table 2. ZIKV model selection in BEAST using PS/SS MLE to investigate co-circulation of dengue and South American–origin Zika viruses, Pakistan, 2021–2022*

Clock	Tree	PS log MLE	SS log MLE	Rank
Relaxed	Skyline	−41538.701364	−41544.221456	1
Random	Skyline	−41564.818442	−41572.615305	2
Relaxed	Constant	−41582.924403	−41586.979714	3
Relaxed	Exponential	−41591.147698	−41594.833115	4
Random	Skyride	−41613.561723	−41615.591782	5
Relaxed	Skyride	−41611.693660	−41615.931166	6
Random	Constant	−41626.134183	−41625.191906	7
Strict	Skyline	−41628.226215	−41635.039381	8
Strict	Exponential	−41648.922299	−41652.766987	9
Strict	Constant	−41683.036957	−41685.618307	10
Random	Exponential	−41799.307050	−41801.812929	11
Strict	Skyride	−41837.651897	−41842.479273	12

*Path sampling (PS)/stepping stone (SS) marginal likelihood estimation (MLE); ZIKV: Zika virus; BEAST: Bayesian Evolutionary Analysis Sampling Trees (https://beast.community/index.html)

Appendix Table 3. Real time qRT-PCR assay primers and probes to investigate co-circulation of dengue and South American–origin Zika viruses, Pakistan, 2021–2022

Pan-Dengue	Sequence (5′-3′)	5′Fluorophore	3′Quench
**DENV1,2,3-F**	CAGATCTCTGATGAACAACCAACG		
**DENV2-F**	CAGATCTCTGATGAATAACCAACG		
**DENV3-F**	CAGATTTCTGATGAACAACCAACG		
**DENV4-F**	GATCTCTGGAAAAATGAAC		
**DENV1,3-R**	TTTGAGAATCTCTTCGCCAAC		
**DENV2-R1**	AGTTGACACGCGGTTTCTCT		
**DENV2-R2**	AGTCGACACGCGGTTTCTCT		
**DENV4-R**	AGAATCTCTTCACCAACC		
**DENV1-Pr**	pdCpdUpdCGpdCGpdCGpdUpdUpdUpdCAGpdCApdUApdUA	FAM	BHQ-1 plus
**DENV2-Pr**	pdCpdUpdCpdUpdCGpdCGpdUpdUpdCAGpdCApdUApdU	FAM	BHQ-1 plus
**DENV3-Pr**	pdCpdUpdCpdUpdCApdCGpdUpdUpdCAGpdCApdUApdUpdUG	FAM	BHQ-1 plus
**DENV4-Pr**	pdCpdUpdCApdCGpdCGpdUpdUpdCAGpdCApdUApdU	FAM	BHQ plus
**Pan-Zika**			
**ZIKV-F**	CAGCTGGCATCATGAAGAAYC		
**ZIKV-R1**	CACTTGTCCCATCTTCTTCTCC		
**ZIKV-R2**	CACCTGTCCCATCTTTTTCTCC		
**ZIKV-Pr**	CYGTTGTGGATGGAATAGTGG	CIV550	BHQ-1
**Zika duplex**			
**ZIKV 835**	TTGGTCATGATACTGCTGATTGC		
**ZIKV-911c**	CCTTCCACAAAGTCCCTATTGC		
**ZIKV-1086**	CCGCTGCCCAACACAAG		
**ZIKV-1162c**	CCACTAACGTTCTTTTGCAGACAT		
**ZIKV-860-Pr**	CGGCATACAGCATCAGGTGCATAGGAG	FAM	BHQ-1 plus
**ZIKV-1107-Pr**	AGCCTACCTTGACAAGCAGTCAGACACTCAA	FAM	BHQ-1 plus

qRT-PCR: Quantitative reverse transcription polymerase chain reaction; F: Forward; R: Reverse; Pr: Probe

Appendix Table 4. Summary of patient metadata to investigate co-circulation of dengue and South American–origin Zika viruses, Pakistan, 2021–2022

Patient	Sex	Age, y	Fever	Chills	Headache	Myalgia/arthralgia	Nausea	Rash	Hospital diagnosis
**A**	F	46–50	Y	N	Y	Y	Y	Y	FUO
**B**	M	16–20	Y	Y	Y	Y	N	N	FUO
**C**	F	11–15	Y	Y	Y	Y	Y	N	FUO
**D**	F	31–35	Y	N	Y	Y	N	N	Fever, UTI
**E**	M	56–60	Y	Y	N	Y	Y	Y	Dengue fever
**F**	F	26–30	Y	Y	N	Y	Y	Y	FUO
**G**	M	NA	N	N	N	N	N	N	NA

N, no, NA, not available; FUO: fever of unknown origin; UTI: urinary tract infection; Y: yes

Appendix Table 5. Key study time points for enrolled patients following day 1 symptom onset to investigate co-circulation of dengue and South American–origin Zika viruses, Pakistan, 2021–2022

Patient	Symptom onset	Hospital admission	Sample collection	Hospital discharge	Source
**A**	Nov 2021	Day 7	Day 9	Day 11	FELTP
**B**	Nov 2021	Day 7	Day 10	Day 9	FELTP
**C**	Nov 2021	Day 6	Day 8	Day 10	FELTP
**D**	Nov 2021	Day 4	Day 9	Day 9	AKU
**E**	Nov 2021	Day 4	Day 7	Day 8	AKU
**F**	Nov 2021	NA	Day 6	NA	AKU
**G**	Nov 2021	NA	NA	NA	FELTP

FELTP: Field Epidemiology Laboratory Training Program; AKU: Aga Khan University Hospital; NA, not applicable

Appendix Table 6. Top Genome Detective viral metagenomics sequence identification calls per sample to investigate co-circulation of dengue and South American–origin Zika viruses, Pakistan, 2021–2022

Patient	Strain Name	Reads	Depth	Nt ID	Aa ID	Coverage
A	Dengue virus 2	79318	3852.39	0.94	0.97	21.51
B	Pegivirus hominis	1018151	19416.41	0.90	0.97	63.92
B	Dengue virus type 2	1035353	25586.34	0.92	0.97	42.14
C	Dengue virus type 2	16600	198.32	0.92	0.97	98.79
C	Human endogenous retrovirus K113	155	11.39	0.92	0.90	16.53
C	Bracoviriform glomeratae	6	4.88	0.77	0.91	31.22
C	Harvey murine sarcoma virus	2	1.37	0.84	0.92	14.24
C	Harvey murine sarcoma virus	2	1.13	0.77	0.95	18.36
D	Dengue virus 2	120478	1965.41	0.92	0.97	63.25
E	Dengue virus 2	51778054	565067.79	0.92	0.97	97.42
E	Zika virus	2956	32.71	1.00	1.00	95.32
E	SARS-related coronavirus	155	2.79	1.00	0.98	21.21
E	Sinsheimervirus phiX174	28	1.38	1.00	0.99	42.80
F	Zika virus	4573670	50188.99	1.00	1.00	95.71
G	Dengue virus 2	19125	1270.28	0.92	0.96	15.08
G	Betainfluenzavirus influenzae	1040	965.27	1.00	0.97	9.49

Nt ID: Nucleotide identity; Aa ID: Amino acid identity

Appendix Table 7. Sample metadata for patient real time qRT-PCR and serology screening to investigate co-circulation of dengue and South American–origin Zika viruses, Pakistan, 2021–2022

Patient	Age, y	Gender	Collection month	Days of symptoms	Collection day*
**1**	41–45	M	May 2022	7	1
**2**	51–55	M	May 2022	3	1
**5**	36–40	M	May 2022	5	28
**6**	21–25	M	May 2022	2	28
**2**	51–55	M	Jun 2022	3	28
**3**	71–75	M	Jun 2022	7	1
**4**	6–10	M	Jun 2022	3	1
**7**	26–30	F	Jun 2022	2	28
**8**	31–35	M	Jun 2022	3	28
**9**	16–20	F	Jun 2022	3	28
**3**	71–75	M	Jul 2022	7	28
**4**	6–10	M	Jul 2022	3	28
**10**	21–25	M	Sep 2022	3	28
**11**	51–55	F	Sep 2022	6	28
**12**	41–45	M	Sep 2022	2	28
**13**	26–30	M	Oct 2022	4	28

qRT-PCR: Quantitative reverse transcription polymerase chain reaction; *Day 1 acute phase, day 28 convalescent phase

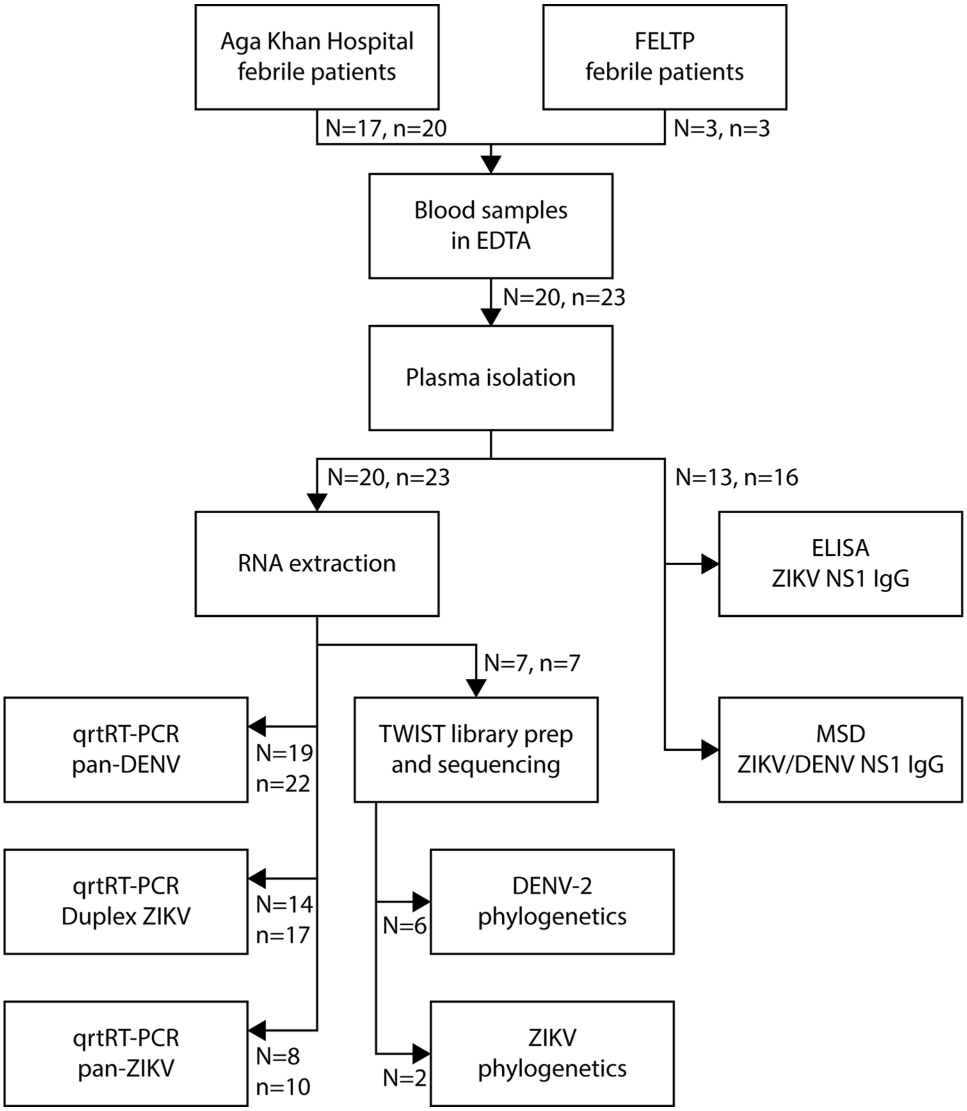

Appendix Figure 1. Study enrollment and design for identification of co-circulating dengue and South American–origin Zika viruses, Pakistan, 2021–2022. Twenty patients presenting with febrile illness at 2 hospitals in Karachi, Pakistan, in November 2021–October 2022 were enrolled for opportunistic surveillance. Blood was processed to test for (1) evidence of viral RNA using quantitative real-time reverse transcription polymerase chain reaction (qrtRT-PCR) and sequencing using the Twist pan-viral metagenomics approach, and (2) serologic evidence of recent arbovirus infection by ELISA and Meso Scale Discovery (MSD) in plasma IgG antibodies. N, patient count; n, sample count; FELTP, Field Epidemiology Laboratory Training Program; EDTA, ethylenediaminetetraacetic acid; ZIKV, Zika virus; DENV, dengue virus; NS1, nonstructural protein 1.

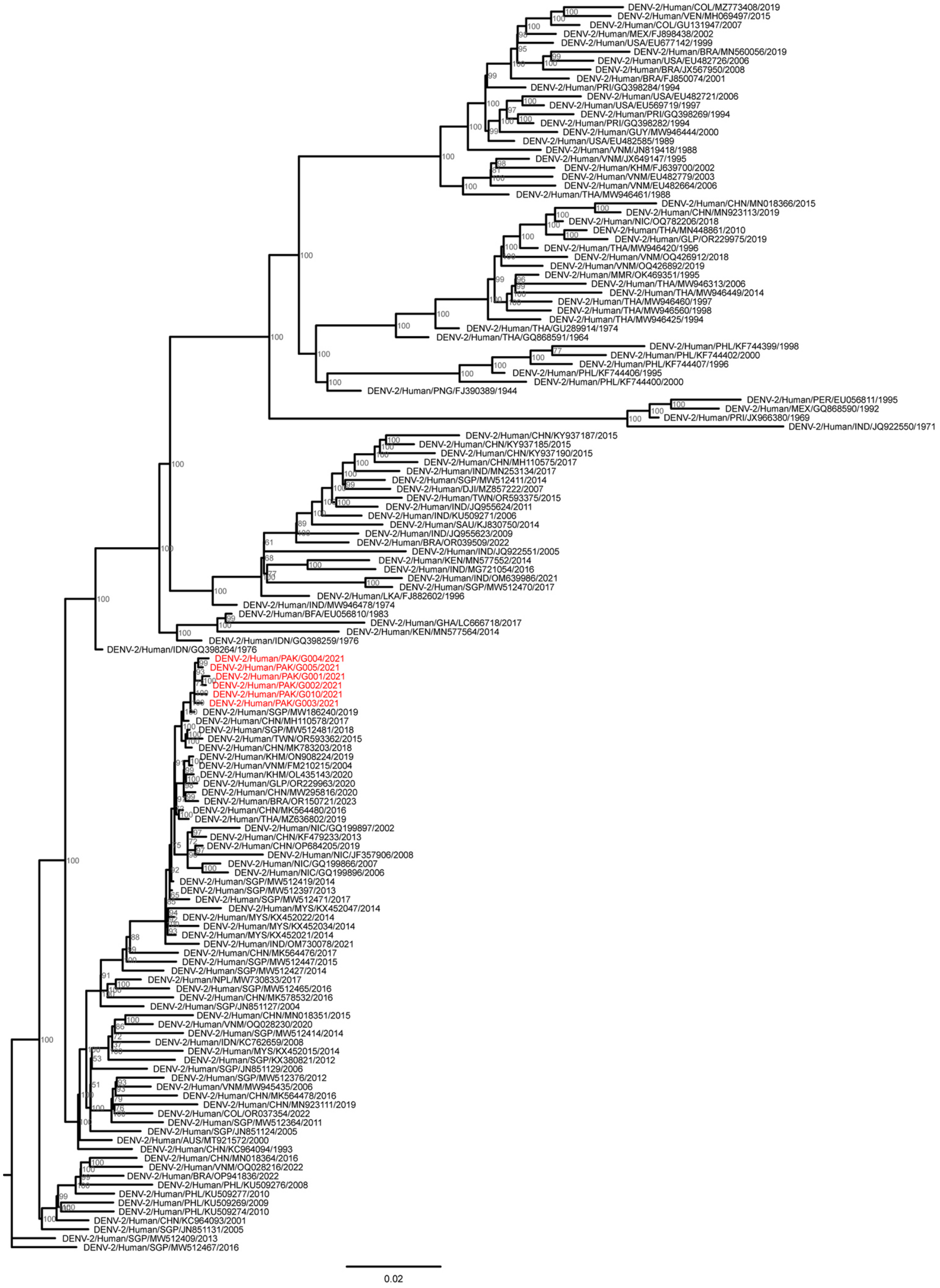

Appendix Figure 2. Dengue virus serotype 2 (DENV-2) maximum likelihood tree with Pakistan-origin viruses for identification of co-circulating dengue and South American–origin Zika viruses, Pakistan, 2021–2022. Final DENV-2 sequence set (n=150) phylogenetic tree produced with IQ-TREE using a GTR+F+R3 model with 1001 ultrafast bootstraps. Posterior probabilities shown in grey; newly described viruses shown in red.

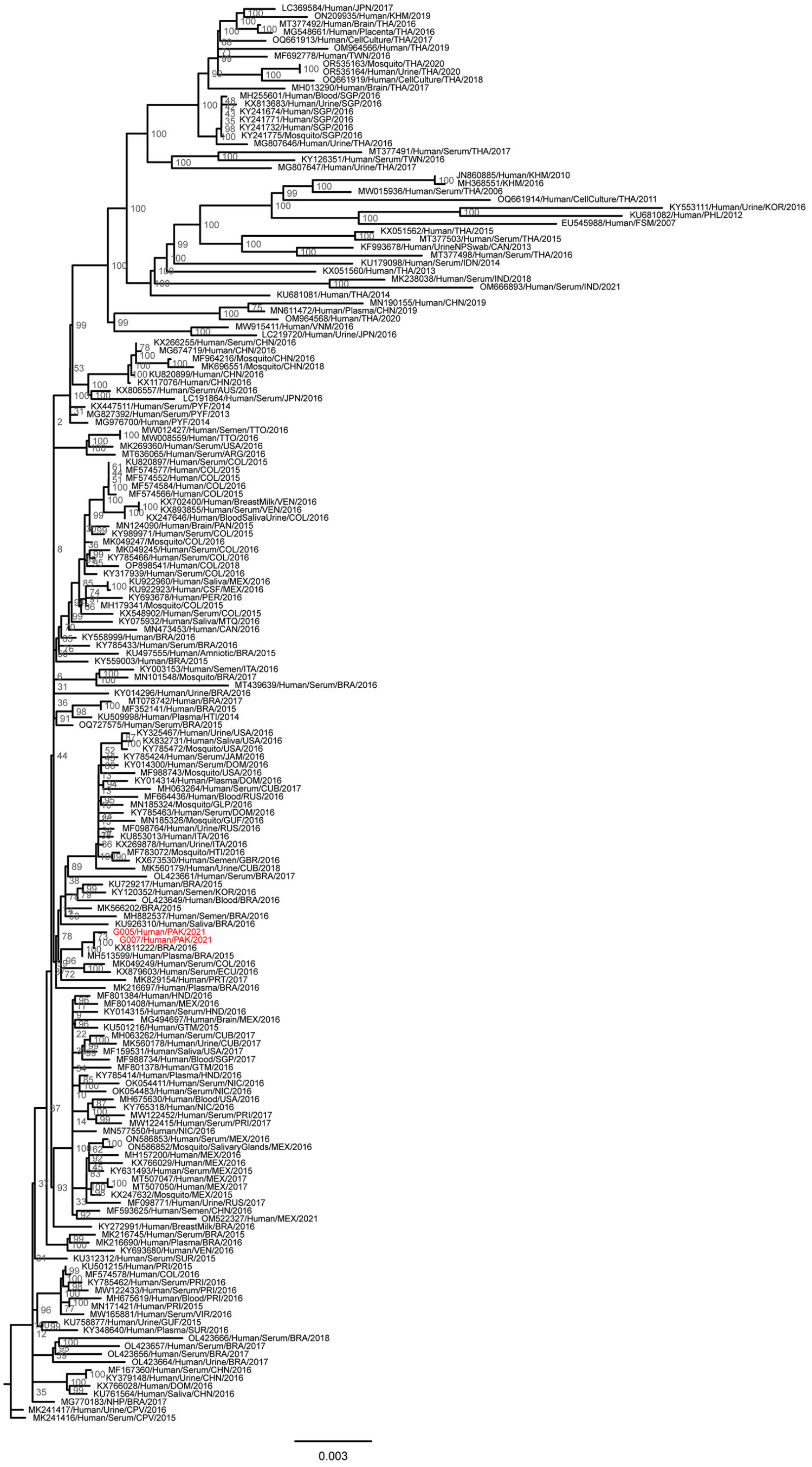

Appendix Figure 3. Zika virus (ZIKV) maximum likelihood tree with Pakistan-origin viruses for identification of co-circulating dengue and South American–origin Zika viruses, Pakistan, 2021–2022. Final ZIKV sequence set (n=176) phylogenetic tree produced with IQ-TREE using a GTR+F+R3 model with 1001 ultrafast bootstraps. Posterior probabilities shown in grey; newly described viruses shown in red.

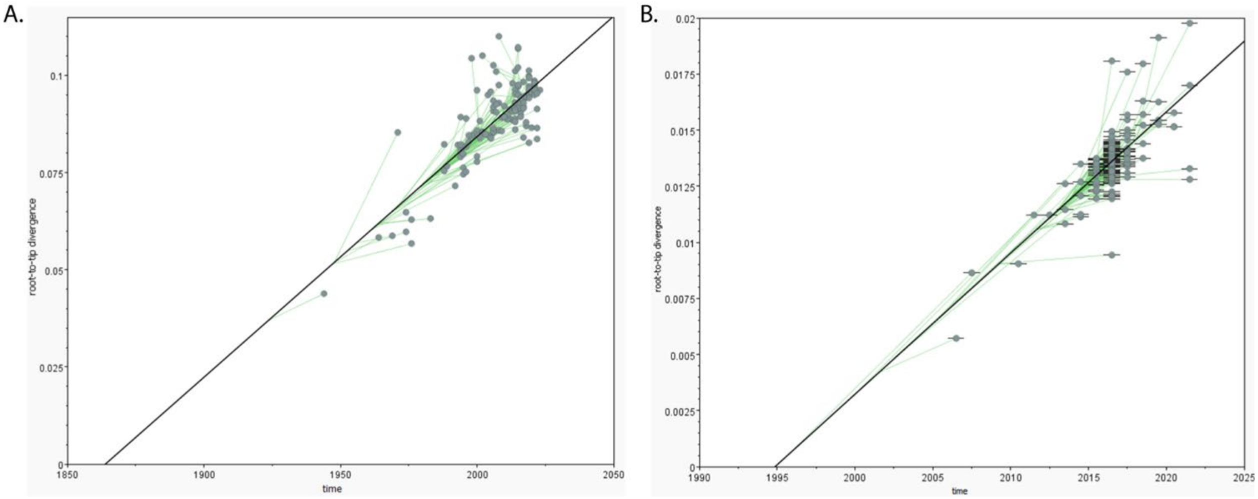

Appendix Figure 4. Molecular clock testing of datasets including Pakistan-origin viruses for identification of co-circulating dengue and South American–origin Zika viruses, Pakistan, 2021–2022. Final maximum likelihood trees for (A) dengue virus (DENV) and (B) Zika virus (ZIKV) were tested for appropriateness of molecular clock modelling with TempEst using best-fitting root. Correlation coefficients are 0.81 (R^2=0.66) for DENV and 0.76 (R^2=0.58) for ZIKV.

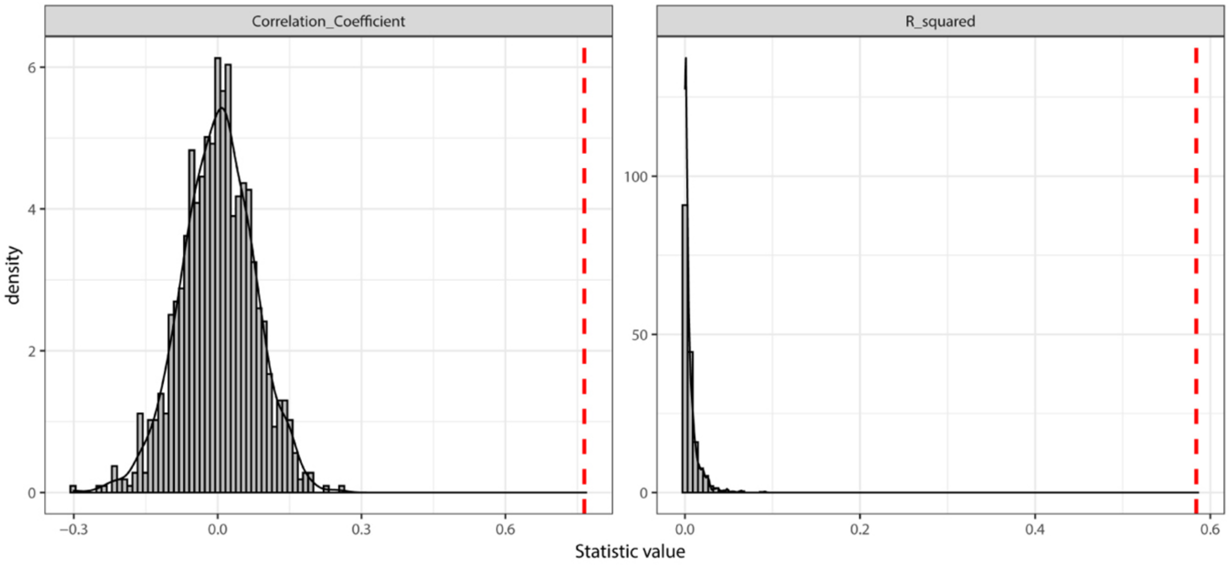

Appendix Figure 5. Molecular clock testing for Zika virus (ZIKV) sequences against a null distribution for identification of co-circulating dengue and South American–origin Zika viruses, Pakistan, 2021–2022. A null set of 1000 randomly scrambled genome-metadata was processed through IQ-TREE (m=GTR+F+R3, bb=1001) and TempEst (best-fitting root) and distribution of correlation coefficient and R^2 values plotted in grey. Red dashed lines are values of observed data from Appendix Figures 3 and 4.

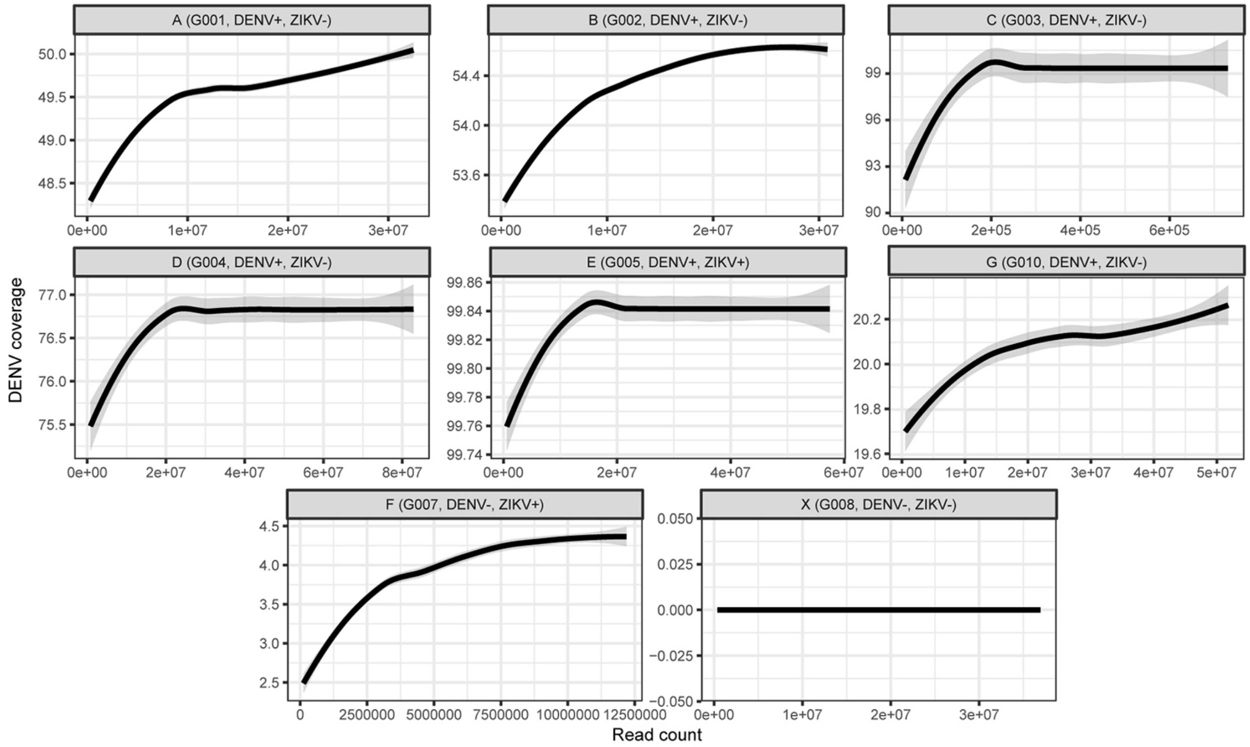

Appendix Figure 6. Dengue virus (DENV) genome coverage using the Twist platform for identification of co-circulating dengue and South American–origin Zika viruses, Pakistan, 2021–2022. Total reads per sample were mapped to a combined human genome with DENV-1-4 reference. All mapped and unmapped reads were randomly subsampled to represent increasing proportions of total available reads in 1% increments. DENV-2 genome coverage from mapped reads per sample-percent were fit with a locally estimated scatterplot smoothing regression, with 95% confidence interval shaded in grey. Sample X was prepared in the same manner from a patient without viral fever. ZIKV, Zika virus.

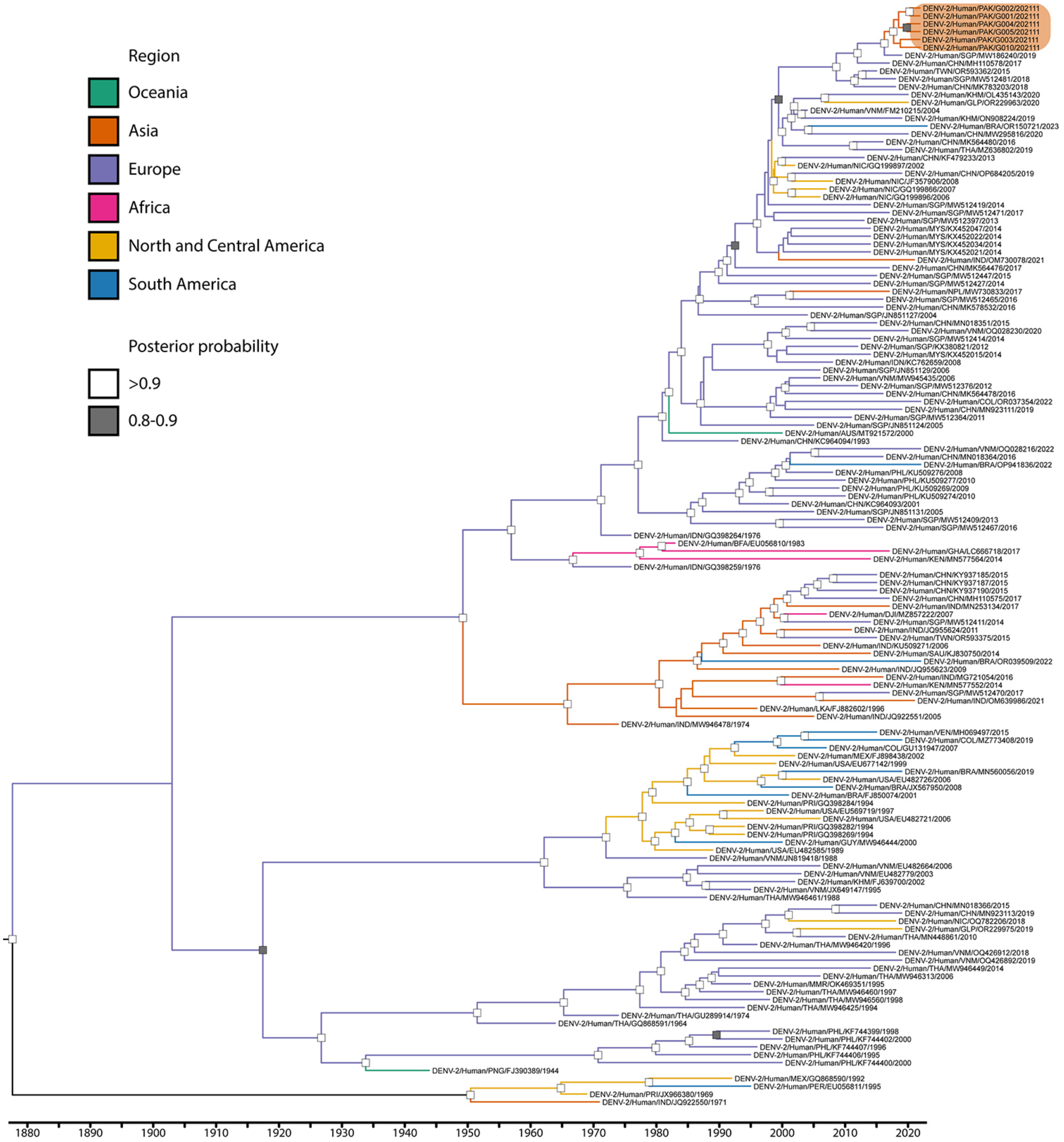

Appendix Figure 7. Bayesian phylogeographic analysis of patient plasma infected with dengue virus serotype 2 (DENV-2) with sequence metadata for identification of co-circulating dengue and South American–origin Zika viruses, Pakistan, 2021–2022. Bayesian evolutionary analysis sampling trees (BEAST) time-aware maximum clade credibility tree describing inferred genetic lineage of global dengue virus strains, colored by observed and estimated geographic origin. Branch backbones are colored when called with >70% confidence by Augur.

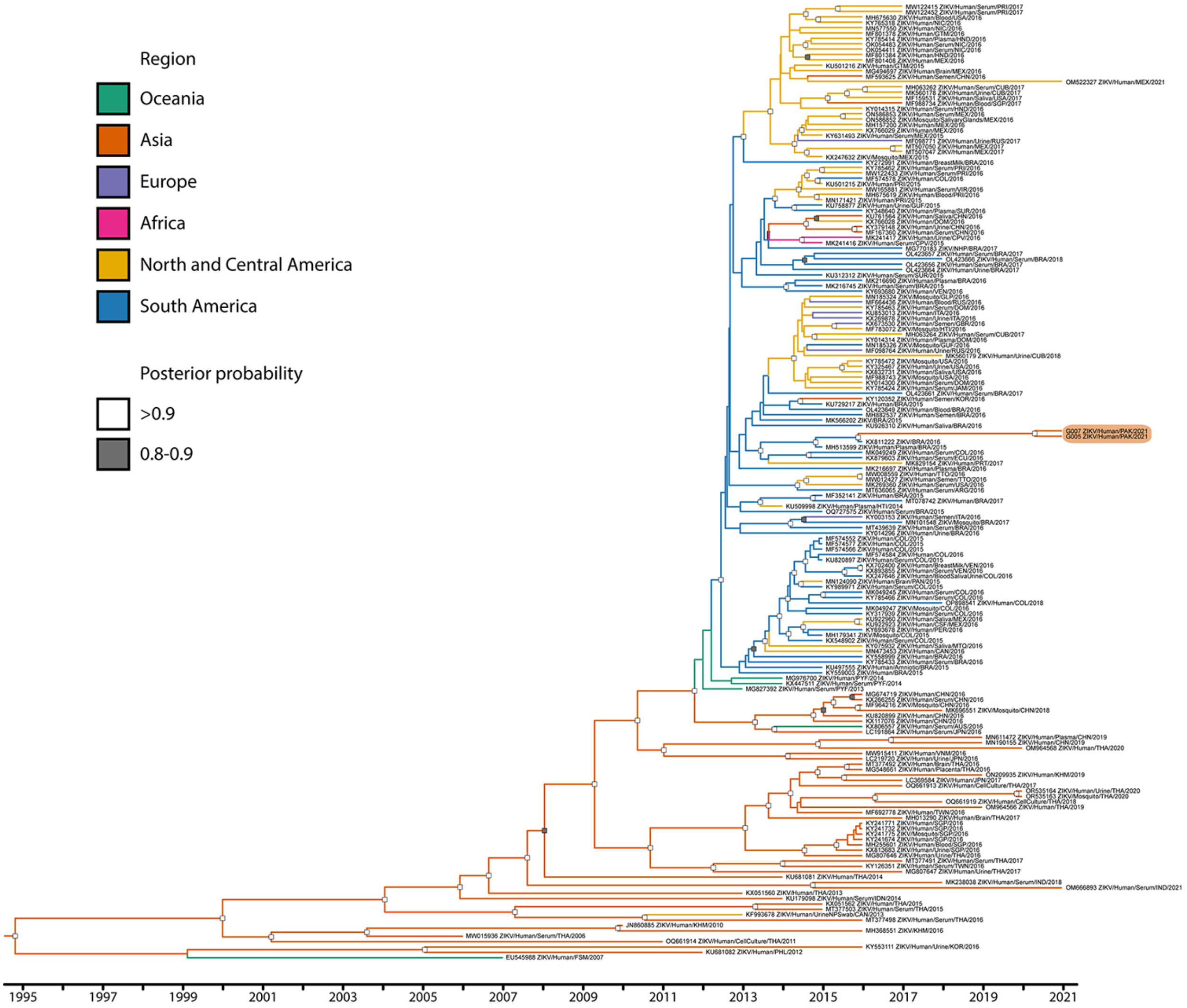

Appendix Figure 8. Bayesian phylogeographic analysis of patient plasma infected with Zika virus (ZIKV) with sequence metadata for identification of co-circulating dengue and South American–origin Zika viruses, Pakistan, 2021–2022. Bayesian evolutionary analysis sampling trees (BEAST) time-aware maximum clade credibility tree describing inferred genetic lineage of global Asian-lineage ZIKV, colored by observed and estimated geographic origin. Branch backbones are colored when called with >70% confidence by Augur.

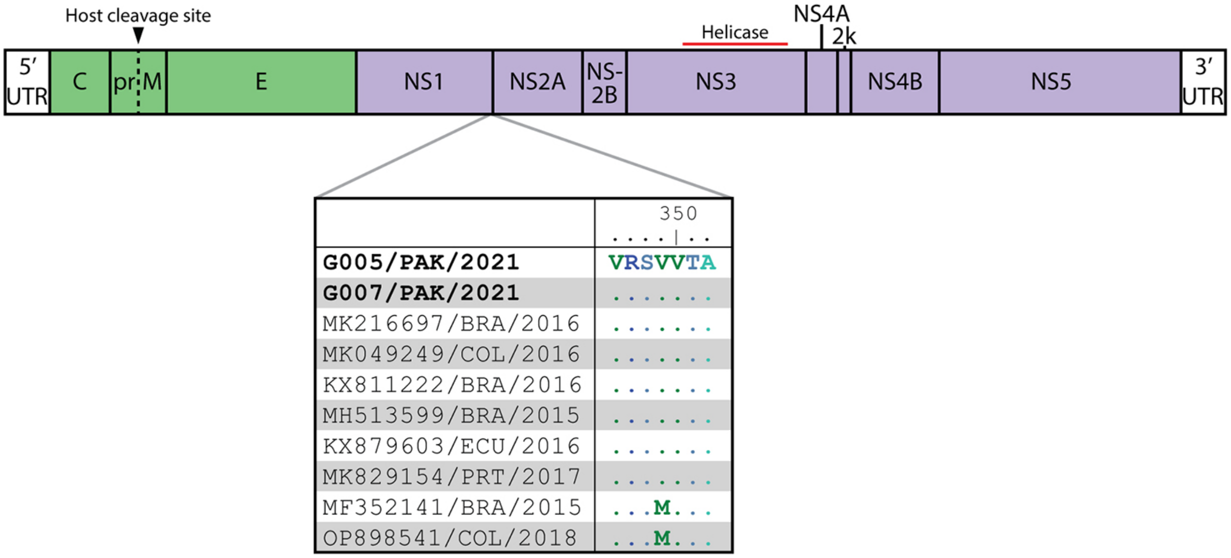

Appendix Figure 9. Intraclade Zika virus (ZIKV) amino acid changes encoded in the nonstructural protein 1 (NS1) gene for identification of co-circulating dengue and South American–origin Zika viruses, Pakistan, 2021–2022. ZIKV sequences were selected from the same clade and subclade as newly described Pakistan-origin viruses and aligned to G005/PAK/2021. Two additional South American-origin viruses were selected outside of the subclade for comparison. Identical amino acid residues are shown as dots. G005/PAK/2021 corresponds to patient E and G007/PAK/2021 corresponds to patient F. M349V distinguishes the Pakistan-inclusive Brazilian subclade from other South American circulating viruses. 2K, protein 2K; C, capsid; E, envelope; NS2A, nonstructural protein 2A; NS2B, nonstructural protein 2B; NS3, nonstructural protein 3; NS4A, nonstructural protein 4A; NS4B, nonstructural protein 4B; NS5, nonstructural protein 5; prM, precursor membrane protein; UTR, untranslated region.
